# Development of a data-driven method for assessing health and welfare in the most common livestock species in Switzerland: The Smart Animal Health project

**DOI:** 10.3389/fvets.2023.1125806

**Published:** 2023-03-28

**Authors:** Beat Thomann, Hanno Würbel, Thibault Kuntzer, Christina Umstätter, Beat Wechsler, Mireille Meylan, Gertraud Schüpbach-Regula

**Affiliations:** ^1^Veterinary Public Health Institute, Vetsuisse Faculty, University of Bern, Bern, Switzerland; ^2^Animal Welfare Division, Vetsuisse Faculty, University of Bern, Bern, Switzerland; ^3^Identitas AG, Bern, Switzerland; ^4^Research Division on Competitiveness and System Evaluation, Agroscope, Ettenhausen, Switzerland; ^5^Thünen Institute of Agricultural Technology, Braunschweig, Germany; ^6^Centre for Proper Housing of Ruminants and Pigs, Federal Food Safety and Veterinary Office, Agroscope, Ettenhausen, Switzerland; ^7^Clinic for Ruminants, Vetsuisse Faculty, University of Bern, Bern, Switzerland

**Keywords:** health indicators, welfare indicators, monitoring, farm animals, animal-based indicators, machine learning, data integration

## Abstract

Improving animal health and welfare in livestock systems depends on reliable proxies for assessment and monitoring. The aim of this project was to develop a novel method that relies on animal-based indicators and data-driven metrics for assessing health and welfare at farm level for the most common livestock species in Switzerland. Method development followed a uniform multi-stage process for each species. Scientific literature was systematically reviewed to identify potential health and welfare indicators for cattle, sheep, goats, pigs and poultry. Suitable indicators were applied in the field and compared with outcomes of the Welfare Quality^®^ scores of a given farm. To identify farms at risk for violations of animal welfare regulations, several agricultural and animal health databases were interconnected and various supervised machine-learning techniques were applied to model the status of farms. Literature reviews identified a variety of indicators, some of which are well established, while others lack reliability or practicability, or still need further validation. Data quality and availability strongly varied among animal species, with most data available for dairy cows and pigs. Data-based indicators were almost exclusively limited to the categories “Animal health” and “Husbandry and feeding”. The assessment of “Appropriate behavior” and “Freedom from pain, suffering, harm and anxiety” depended largely on indicators that had to be assessed and monitored on-farm. The different machine-learning techniques used to identify farms for risk-based animal welfare inspections reached similar classification performances with sensitivities above 80%. Features with the highest predictive weights were: Participation in federal ecological and animal welfare programs, farm demographics and farmers' notification discipline for animal movements. A common method with individual sets of indicators for each species was developed. The results show that, depending on data availability for the individual animal categories, models based on proxy data can achieve high correlations with animal health and welfare assessed on-farm. Nevertheless, for sufficient validity, a combination of data-based indicators and on-farm assessments is currently required. For a broad implementation of the methods, alternatives to extensive manual on-farm assessments are needed, whereby smart farming technologies have great potential to support the assessment if the specific monitoring goals are defined.

## Introduction

Assessing and improving animal health and welfare, along with using novel possibilities provided by the advancing digitalization in the animal health sector, are focal points of the Swiss Animal Health Strategy 2022+ (1). In this context, animal health means more than absence of diseases and injuries, since, besides the health of the animals, the Swiss Animal Welfare Act also aims at protecting the dignity and wellbeing of the animals (2).

In order to verify the effectiveness of measures implemented to improve animal health and welfare, a method is needed to objectively assess health and welfare at the farm level. Many different approaches are described in the literature. Earlier approaches mainly assessed husbandry and environment (so-called resource-based welfare indicators). Examples are the Animal Needs Index 35 developed in Austria (3) or the Animal Welfare Index 200 developed in Germany (4). These methods are relatively easy to employ. However, their validity for assessing health and welfare is limited, as important aspects are not considered. Other methods narrowly focus on specific aspects of health, such as the BioCheck developed in Belgium, which assesses the biosecurity of a farm (5). Modern methods combine animal-based and resource- or management-based indicators of health and welfare. In the Welfare Quality^®^ (WQ) project, a multinational research project funded by the European Union, welfare assessment methods and corresponding protocols were developed for cattle, pigs and poultry (6, 7). In a follow-up project, similar protocols were also developed for sheep (8) and goats (9). The advantage of these protocols is that they are comprehensive and include assessments of both the animal and its husbandry and environment. The disadvantage of these methods is that they require farm visits and time consuming manual on-farm data collection. Alternatively, data that are routinely collected about animals and animal husbandry practices could provide valuable sources of information on animal health and welfare. Examples for data-based methods are the Animal Health Barometer (10), which describes the evolution of the health status at population level or the MulTiViS method (11), which focuses on secondary data usage for health monitoring in pigs. With the increasing availability of electronic records and extensive databases, the potential for such data-based methods is rapidly increasing. This also applies to “smart farming” data from Precision Livestock Farming (PLF) technologies, which might be used to monitor animal health and welfare (12).

However, there is a lack of scientific studies that systematically assessed the reliability and feasibility of the methods mentioned above and combined them with on-farm assessments to determine their validity as measures of animal health and welfare. Various recently launched initiatives and research projects to establish methods for animal health and welfare monitoring or labels in different countries, such as “Bedre Dyrevelfaerd” in Denmark (13), “Nationales Tierwohl Monitoring” (14) and “Tierwohlkennzeichen” (15) in Germany or “Classyfarm” in Italy (16), also highlight that country-specific approaches are needed to consider the diversity of production systems, data management and availability, as well as acceptance of the method(s) by stakeholders.

The aim of this project was to develop a method to assess animal health and welfare for different livestock species in Switzerland, by integrating animal-based indicators with data-driven metrics that may serve as proxies for the health and welfare status at the level of individual farms, groups of farms, and the Swiss livestock population as a whole. Additionally, the availability and potential of PLF technologies for the assessment of health and welfare was examined. Successful implementation of the method should then allow for: (i) monitoring changes in the health status of livestock populations and in individual farms over extended time periods; (ii) assessing the effectiveness of measures taken to improve animal health and welfare; (iii) identifying farms with particularly good animal health in view of promoting them with financial incentives; and (iv) implementing risk-based animal welfare inspections.

This manuscript provides an overview of the work carried out as part of the Smart Animal Health (SAH) research project, outlines the systematic process of method development, highlights specific results and discusses certain aspects of the overall project. Detailed results will be, and for some parts already have been published in separate publications.

## Materials and methods

### Method development

**Figure 1:**
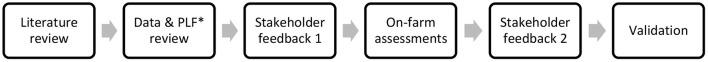
Multi-stage development process of the Smart Animal Health method used to assess herd-level health and welfare status. *PLF, Precision Livestock Farming.

The SAH method was developed in a uniform multi-stage process for all included animal species and categories (Figure 1). These were dairy cows, veal calves, sheep, goats, sows, fattening pigs, broilers and laying hens (the latter two hereinafter referred to as poultry).

First, a context analysis was conducted, consisting of a review of scientific literature, data availability and the potential of existing PLF technologies. For better readability, the data and PLF review will be discussed in separate sections. In order to determine the current state of research regarding existing indicators for assessing animal health and welfare, a systematic literature research was carried out for each animal species and category involved. For literature identification, search queries were made in the scientific databases PubMED, Web of Science, Scopus, CAB Direct and Science Direct, and identified publications were analyzed based on the PRISMA guidelines (17). Detailed methods of the literature review for the different animal species are described in Gebhardt-Henrich and Schlapbach (18), Lutz et al. (19), Minning et al. (20), and Zufferey et al. (21). Subsequently, a preliminary set of promising indicators was compiled and discussed with stakeholders. The number of external stakeholders per workshop varied between 16 and 28. For cattle (n_workshop1_ = 22; n_ws2_ = 28), pigs (n_ws1_ = 18; n_ws2_ = 23) and poultry (n_ws1_ = 16; n_ws2_ = 23) there were two rounds of stakeholder workshops each, one before the farm visits and one afterwards. With a few exceptions, most of the participants and organizations in the second workshop were the same as in the first workshop. For sheep and goats there was only one workshop (n_ws_ = 17). Stakeholders from the relevant sectors participated in the workshops, including people from industry associations, producers, animal transport companies, livestock marketers, processors, retailers, veterinarians, universities, Swiss Veterinary Service and Swiss Animal Welfare Protection. Based on stakeholder feedback and relevance regarding Swiss production conditions, suitable indicators were determined and assigned to four categories, based on the Swiss Animal Welfare Act (2): (i) “Animal health”, (ii) “Husbandry and feeding”, (iii) “Appropriate behavior” and (iv) “Freedom from pain, suffering, harm and anxiety”.

To assess the feasibility of the method, farm visits were carried out in a next step, and the developed SAH method was applied in the field with an elaborated specific set of indicators per animal category. Based on a convenience sample, a total of 35 dairy cattle, 31 veal calf, 27 pig and 17 poultry farms were visited and data on SAH indicators were collected. For sheep and goats, no farm visits were conducted, as the context analysis had indicated that the development of a data-driven method for these species was not feasible at this stage. During the farm visits, the WQ protocol was also carried out to assess the animal health and welfare status of a given farm, which later served as reference value for comparison with results based on SAH indicators. For laying hens, the MTool (22) was used as reference method, because this tool is more widely used for welfare assessment in laying hens in Switzerland than the WQ protocol. Following the farm visits, a second round of stakeholder workshops was held. These workshops were conducted online, and feedback was collected using Mentimeter (23), a web-based tool to anonymously capture real-time participant feedback and stimulate discussion. Results of the field studies were presented and discussed, and the method was further refined (e.g., regarding categorization or importance of individual indicators). Finally, outcomes from the SAH and WQ methods were compared and assessed for consistency using descriptive and inferential statistical methods. Within the SAH method, a dual approach was chosen in order to account for dependencies and restrictions in the scoring and integration of the individual indicators: On the one hand, a purely descriptive statistical analysis of the scores was carried out in view of benchmarking; on the other hand, individual indicators were scored by means of thresholds with target and alarm values. For the benchmarking, indicator values were standardized by means of Z-transformation and compared to the other farms serving as reference population. The 25 and 75% quartile served as cut-offs and scores were categorized into “lowest 25%”, midfield (25–75%) or “best 25%” (>75%). The assessment against target and alert values was mainly based on thresholds retrieved from the literature (24–26). If literature data were insufficient or not applicable for Swiss production systems, thresholds were adapted or defined by informal expert consultation.

### Data context

In the data context analysis, existing databases and information systems were assessed within the framework of animal health and welfare and examined for the availability of data for possible indicators. Data sources containing potential indicators can be allocated into three categories: (i) public data sources, where content and access are regulated under public law, (ii) private data sources that are owned by farmers and/or private organizations, and (iii) so-called “on-farm” data, which refers to data that do not yet exist in a database and must be collected on the farm (Table 1). Consequently, “data-based indicators” were defined as indicators for which data can be retrieved from existing public or private data sources and “on-farm indicators” as indicators for which data must be obtained “on-farm”.

**Table 1:** Overview of the categorization of selected data sources and indicators with description of their availability, coverage and usefulness for assessing animal health and welfare in Swiss livestock farms.

	**Public data^a^**	**Private data**	**On-farm**
Data sources	E.g., animal movement database (TVD), agricultural policy information system (AGIS), laboratory information system (ALIS), information system for control data (ACONTROL)	E.g., breeding associations databases, slaughterhouse records, integrator's production and health data	None; must be assessed through farm visits
Indicators	E.g., mortality, farm demographics, participation in animal welfare programmes	E.g., somatic cell counts, milk yield, weight gain, treatment incidences	E.g., lameness, body condition score, qualitative behavior assessment
Data availability	High	Limited	None
Coverage	High	Mid-high	Very low
Usefulness	Low-mid	Mid-high	High
Comments	Different granularity and availability depending on species/category	Different availability depending on owner and membership	On-farm data collection is very time consuming

a Access and content regulated by public law.

The most relevant public Swiss data sources are the animal movement database (TVD), the agricultural policy information system (AGIS), the laboratory information system (ALIS, recently renamed to ARES), the information system for control data (ACONTROL) which contains records on animal welfare inspections, the information system on antibiotics in veterinary medicine (IS-ABV), and the meat inspection database (FLEKO). The IS-ABV was introduced in 2019 and contains the antibiotic prescriptions according to the Therapeutic Products Act (27), with information on oral group therapies, individual animal treatments as well as the dispensing of veterinary medicinal products in stock. The updated version of FLEKO was launched in 2020 and contains information on ante-mortem inspection of live animals and condemnation of partial and whole carcasses. As IS-ABV and FLEKO were only introduced shortly before the start of the SAH project, they could not be considered in the analyses because of data quality issues. Private data sources included for example data from breeding associations, abattoir data on carcass evaluations, data from animal health services, records in electronic treatment journals, performance data, data from producer organizations and integrators (especially in broiler and veal calf production), parts of the mandatory milk quality analyses, as well as other records from the farmers or their veterinarians. The “on-farm” data include specific data on animal welfare evaluation such as qualitative behavior assessment or indicators for “Freedom from pain, suffering, harm and anxiety”.

### PLF technologies

A systematic search on available PLF technologies was carried out focusing on automated technologies for real-time monitoring of animals. These systems usually consist of a sensor and smart software, which collect and analyze data of animals and/or their environment (12). The review focused specifically on PLF technologies in the animal health and welfare context that could be used to simplify and improve data collection of on-farm indicators. First, it was determined which PLF systems are commercially available, followed by reviewing which systems or single parameters have been scientifically validated (28). The overviews for the different animal species were based on the “4D4F Technology Warehouse” format (29) structured by the main purpose of the systems (e.g., feeding, activity, lameness, etc.). Subsequently, further compilations of PLF systems (commercially available or in development) were produced to capture specific SAH indicators (30). To examine the potential of PLF systems for health and welfare assessment in livestock, obstacles and challenges in the development of PLF technologies were identified *via* a systematic literature search based on the PRISMA guidelines (17), and a framework for the automated detection of health- and general welfare-related issues was developed as a guideline for future studies (31).

### Risk index

To address the aim of implementing risk-based animal welfare inspections, a purely data-driven approach was chosen based on public databases with high data availability and coverage. Different databases and national registries (i.e., TVD, AGIS, ALIS, ACONTROL) containing information on farm demographics, animal identification, traceability, diagnostic results, and animal welfare inspections were pseudonymised and interconnected. Exploratory data analysis combined with machine learning algorithms [Support vector machine (SVM), Logistic regression, Random forest (RF) and Artificial Neural Network (ANN)] were used to identify relevant proxies for animal health and welfare. Input data (so-called features of the holding) were fed to machine learning algorithms that built a model of classification. Features were derived from extracts of the national databases cited above, like population and mortality by age, participation to additional welfare programs or labels, and geographical information. Features were preprocessed to reduce dimensionality where needed, to transform them into categorical values and standardized. The results of previous on-farm welfare inspections recorded in ACONTROL were used to calibrate a binary welfare index. Farms where 50% or more of the control points were found to be deficient were considered to be “at risk” for animal welfare violations. Different methods, including logistic regression, random forests or artificial neural networks were applied and performances of the different algorithms were assessed through a *k*-fold method. Meta-parameters of the methods were chosen to maximize performances. Performance was assessed using the F_1_-score and similar metrics between the ground truth and the predictions of the algorithms. The training and testing of the algorithms were repeated 1,000 times to be able to assess training variability. Further details on the methodology for the risk index will be described in a separate publication.

## Results

### SAH method

The literature review identified a large number of potential indicators for the respective animal species and categories. For dairy cows, broilers, sheep and goats, detailed results of the reviews have been published (18–21). Some of the identified indicators are already well established and widely used in practice (e.g., mortality rate, lameness score, somatic cell count), while others lack reliability or practicability and/or require further research and validation (e.g., pain assessment, eye condition). Moreover, it was found that some indicators have only limited suitability for Swiss production systems, for example due to smaller herd sizes (when based on proportion of animals) or different welfare regulations (e.g., prohibition of tail docking).

All SAH indicator sets developed for the different livestock categories consisted of data-based indicators (indicators for which data could be retrieved from existing public or private data sources) as well as on-farm indicators for which data had to be collected “on farm”. Depending on animal category, there were considerable differences in the origin and availability of the data. In addition, the number of indicators per category varied between the different animal categories. Table 2 gives an overview of assessed indicators during pig farm visits along with information on data availability, distribution and cut-off values. The validity of the SAH method was evaluated by comparing the outcomes with the WQ or MTool protocols carried out in parallel. It became apparent that certain aspects of animal health could already be assessed with a relatively high degree of validity. If public data, private data and on-farm records can be used, valid estimations can be made for all four defined categories of animal health and welfare. If, on the other hand, only information on data-based indicators are available, the validity decreases significantly and in particular the welfare categories “Appropriate behavior” and “Freedom from pain, suffering, harm and anxiety” can usually only be assessed inadequately or not at all (33). Therefore, for sufficient validity, a combination of data-based and on-farm indicators with direct assessments of the animals is needed.

**Table 2:** Overview of indicators assessed during the pig farm visits (*n* = 27) in addition to the Welfare Quality (WQ) protocol, grouped into the four corresponding categories.

**Category**	**Indicator (unit)**	**Data-based**	**On-farm assessment**	**Median (25th and 75th percentiles)**	**Target value**	**Alarm value**
Animal Health	Antimicrobial usage^a^ (index)	X		0.06 (0;0.1)		
	Lameness (%)^b^		X	3.6 (2,4)	< 1	>3
	Mortality (%)	X		1.3 (0.7;1.8)	< 1.5	>3
	Slaughter findings^c^ (%)	X		0.9 (0;2)		
	Stillbirth (%)	X		6.3 (5,8)	< 5	>10
Husbandry and feeding	Body Condition Score 5^d^ (%)		X	6.5 (0;10)	< 3	>6
	Welfare programmes^e^ (y/n)	X		0.5 (0;1)		
	Biosecurity (-)	(X)		19 (13,25)		
	Temperature and Humidity (-)		X	2.4 (0;4.5)		
	Runts (%)		X	3 (2,4)	< 2	>4
Freedom from pain, suffering harm and anxiety	Pressure sores (%)		X	7.2 (5,10)	< 5	>10
	Shoulder ulcers (%)		X	4 (0;6)	< 5	>10
	Tail lesions (%)	(X)		4.2 (0.75;7)	< 2	>10
	Technopathic lesions (%)		X	1.9 (0;3)	< 2	>5
Appropriate behavior	Herd health management (y/n)	(X)		0.4 (0;1)		
	Supervision frequency (year^−1^)		X	2.5 (2,3)		
	Lying behavior (%)		X	2 (0;5)	< 5	>10
	Soiling (%)		X	13.5 (9,15)	< 5	>10

For each indicator, median, 25th and 75th percentile values are reported, as well as whether information is available from existing databases or on-farm assessment is required. If cut-off values from literature were available, these were also reported.

a Animal treatment index (ATI) (32);

b unit “%” refers to farm-level prevalence;

c prevalence of liver findings, pneumonia, abscess and adhesions;

d prevalence of sows with a BCS of 5;

e participation in animal welfare programs (yes/no).

**Figure 2:**
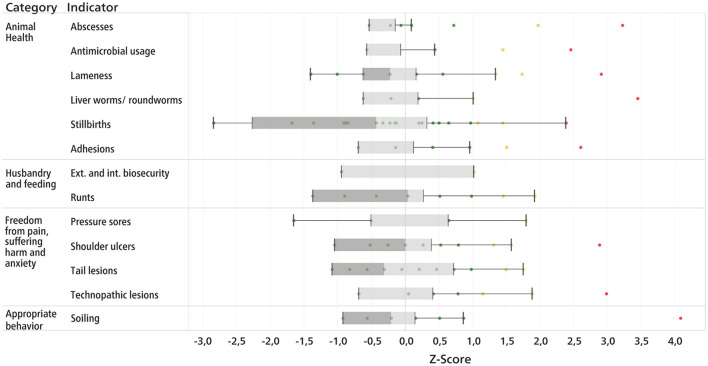
Illustration of benchmarking after Z-transformation for the sow farms (*n* = 27) visited. Lower scores are better than higher scores. Scores below 1 are green, between 1 and 2 are yellow and above 2 are red. The border between dark and light gray indicates the median.

**Figure 3:**
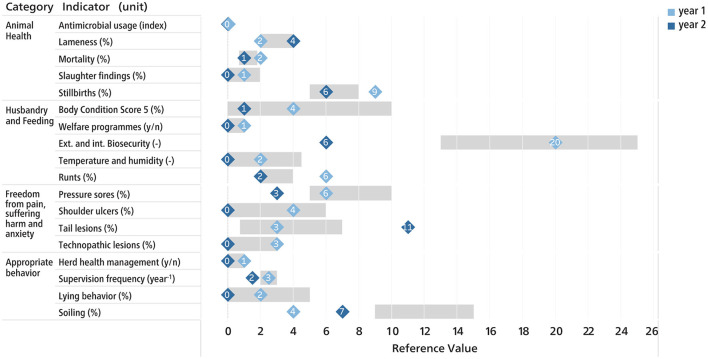
Scoring of an individual sow farm, including benchmarking information of the reference population (27 visited pig farms). The numbers in the squares show the scores of the current (year 1) and the previous (year2) assessment. The gray bar indicates the range between the 25th and 75th percentiles of the reference population. Lower scores are better than higher scores.

**Figure 4:**
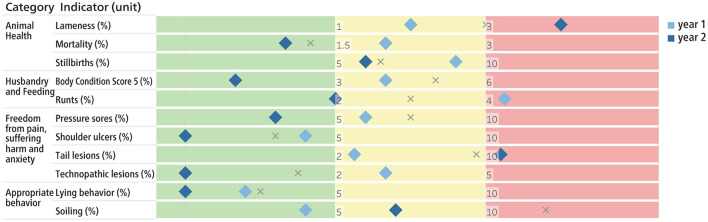
Scoring of the individual indicators for fattening pigs by means of defined target values (between green and yellow area) and alarm values (between yellow and red area). The diamonds show the scores of the current year 1 (dark blue) and the previous year 2 (light blue). The gray cross indicates the median of the reference population.

Several types of possible visualizations of the scores and benchmarking were developed with regard to a future implementation of the method. Figure 2 shows the benchmarking after Z-transformation, as a possible tool for authorities to monitor the health status at population level. Figure 3 shows an illustration for an individual farm, containing benchmarking information compared to other farms. In Figure 4, the scores of the SAH indicators of a single farm are displayed in relation to the corresponding target and alarm thresholds.

### Data review

Most relevant data and information for the assessment of animal health and welfare are generally found in private data sources and data collected “on-farm” (Table 1). However, public data sources are the most accessible to government agencies and also have the widest coverage. In the animal movement database TVD, there are major differences in data quality among animal species. For cattle, there is a high level of detail at the individual animal level. However, there is no specific category for “veal calf”, a common production type in Switzerland, which means that animals intended for fattening cannot be distinguished from dairy calves to be raised as replacement heifers. For pigs, the information is only available at farm and animal group level, and, for poultry, data is limited to the number and age of the animals at the time of entry (stabling) and the production type. The laboratory information system ALIS contains, among other things, test results on notifiable animal diseases. However, these have often limited informative value with regard to the animal health and animal welfare status of a farm. The agricultural policy information system AGIS contains demographic data on farms and information on direct subsidies received for public and ecological services. It mostly does not contain any direct records on animal health, but does contain auxiliary variables (proxies) which can correlate with animal health and welfare indicators.

Exploratory data analyses have shown that, in general, integrating multiple data sources increases their potential compared to using each data source as a stand-alone source. Furthermore, it is important that entire data sets are available for these analyses and that possible parameters do not have to be selected a priori. Since the public databases were pseudonymised, they could be integrated with each other, but not linked to the private databases. The availability and quality of data from private data sources also varies considerably for the different animal categories. For dairy cattle and pigs, various relevant data from existing sources could already be used (with the agreement of the respective data owner). For poultry, potential health and production data are available, but these are mainly owned by integrators, kept in separate data silos by each company, and were only accessible to a very limited extent. The least data is available for veal calves, goats and sheep.

### PLF technologies

The review of PLF technologies, which can be a time-saving and objective alternative to the manual collection of indicators, has revealed two main findings: firstly, the range of PLF technologies varied greatly between different animal categories and, secondly, there is a large discrepancy between scientifically validated and commercially available PLF systems (28, 34). The largest number of PLF technologies is available for dairy cows, both in terms of availability of the number of system types and the number of suppliers within a system type. Fattening pigs and broilers follow in second place, while for sows, laying hens, veal calves, sheep and goats the choice of available PLF technologies that could be used to identify health and welfare issues is very limited (28). Furthermore, the review has shown that there are commercially available PLF technologies for many of the animal-based SAH indicators, however information on the validity of these systems was often not available (30). Based on the systematic literature search, a major weakness for the development of digital support systems was identified which needs to be addressed: It was found that many tools are aimed at identifying specific issues with non-specific indicators, such as activity or feeding. Consequently, most of the current PLF systems, whether in development or commercially available, are potentially able to detect welfare-related changes and rarely more specific issues such as defined diseases or symptoms. To improve the accuracy of PLF technologies, the aim of detection has to be specified and appropriate indicators that are strictly related to the purpose must be chosen (31). The described PLF framework suggests a categorization of the aim of detection into three levels, with each level providing a different degree of information and therefore requiring indicators with different specificity: (i) general welfare, using a large number of non-specific variables; (ii) disease and distress, with a higher degree of information about the cause of an issue; and (iii) defined disease, the most specific decision level, where the aim is to automatically detect signs related to defined diseases (e.g., mastitis).

### Risk index

**Figure 5:**
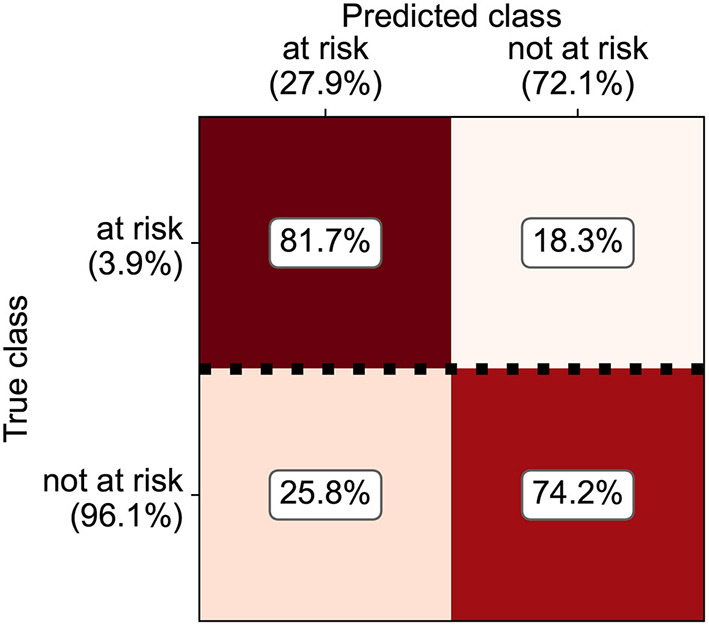
Confusion matrix with classification performances of the random forest model for predicting dairy farms with an increased likelihood for non-compliance with animal welfare law.

**Figure 6:**
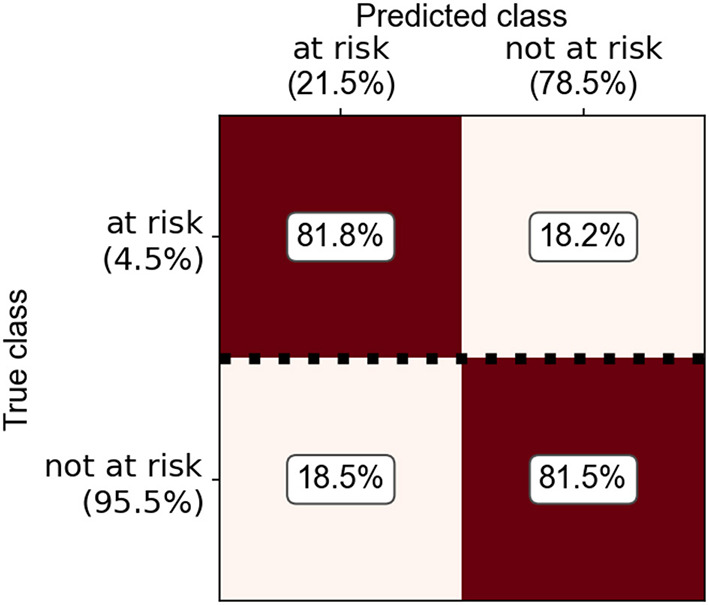
Confusion matrix with classification performances of the random forest model for predicting pig farms with an increased likelihood for non-compliance with animal welfare law.

Due to limited data availability for some species, a risk index could only be estimated for cattle and pig farms. Most of the machine-learning techniques applied reached similar classification performances (Kuntzer et al., submitted). As the explainability of the model was important both for the public acceptance of such a data-driven index as well as for the planning of on-farm welfare inspections, the random forest model was considered to be the most suitable method. Median sensitivity with random forests was 81.7% for cattle farms (Figure 5) and 81.8% for pig farms (Figure 6). Overall, the precision was increased by a factor 3–5 compared to randomly choosing farms for animal welfare inspections. The most important predictors for a lower likelihood of violating animal welfare law were participation in and compliance with federal organic (“ÖLN”) and animal welfare (“BTS” and “RAUS”) programs. Similarly, animal movement reports, notification discipline (farmers are obliged to report events such as births, deaths, leaving or arriving of animals at the farm in the animal movement database TVD within a specified time period) and structural characteristics of the farms, such as the type of husbandry or the standard labor force had high predictive weights.

## Discussion

For the most important livestock categories in Switzerland, a method for assessing animal health and welfare with species-specific indicator sets was developed and evaluated that combines data-based indicators with on-farm indicators. The availability and quality of animal health information varies greatly among the different animal species. For dairy cows and pigs, various data-based indicators already exist and can be used to assess several different categories of animal health. For poultry, good data are available in private data sources, but they were not available for evaluation in this project. For veal calves, sheep and goats, only very few data related to animal health and welfare are systematically and routinely collected. For a high validity of the animal health and welfare assessments, a combination of public and private data with a direct on-farm assessment of animals is currently needed across all animal categories. In particular, the two categories “Appropriate behavior” and “Freedom from pain, suffering, harm and anxiety” cannot be assessed reliably with a purely data-based indicator set. Data availability and quality are crucial for valid estimates, and the predictive power of the indicators sets strongly depends on these factors. Public data sources could be strongly improved by introducing new data variables (e.g., mortality in poultry) or animal categories (e.g., veal calves). Furthermore, fewer free-text fields for data entry (e.g., to describe the reason for a deficiency recorded for a specific control point in an animal welfare inspection) would improve data quality and simplify analyses. The addition of new data sources (e.g., IS-ABV and FLEKO) would further improve the validity of the method. For private data sources, which have been shown to generally contain more valuable data than public sources, accessibility is a key factor. Access to these data can be achieved through data use agreements with data owners. A possible approach to increase the willingness of farmers to share their data would be to pay financial incentives when data is shared and a farm is found to have a particularly good animal health status. However, this only applies if the farmer is the actual data owner and has the right and possibilities to export the data outside the current environment. This is sometimes only possible to a limited extent with commercial data silos owned by private companies. Furthermore, in order to have substantially better access to private data and to avoid biasing datasets that mainly contain data from farms with a particularly good health status, incentives for data-sharing should aim at a higher level, e.g., breeding organizations or label producers. To generate more data for “on-farm” indicators, PLF technologies could be used for time-saving data collection on farms or at key locations such as slaughterhouses. However, to date, most PLF technologies use non-specific indicators and the identification of more specific issues such as defined diseases or symptoms is rarely possible. Yet, for automated data collection based on PLF technologies, a more specific distinction between health-related and welfare-related issues is important (31). In addition, the concept of “iceberg” indicators, with key indicators that effectively summarize many measures of welfare and are easy to understand (35), has proven to be a promising approach for health and welfare assessments with on-farm data (36, 37). With this approach, resources needed for on-farm assessments could be substantially reduced. Two-level assessment protocols, like the AWIN protocol for sheep and goats, represent another alternative (38, 39). At the first level, a screening at the herd-level is carried out with only minimal animal handling. An in-depth second level assessment at animal level is only carried out if the farm is scored below a certain threshold in the first level.

The developed SAH method is subject to several limitations. No farm visits were conducted for sheep and goats. Due to the lack of available data sources, the development of a data-driven method for these species is not feasible at this stage, but only through on-farm assessment using indicators described in the corresponding reviews (20, 21). For the other animal species, the sample size for the farm visits was limited. The focus was on feasibility of the method. However, the samples were too small for robust statistical comparisons, also due to the homogeneity of the farms and production systems in Switzerland. Furthermore, the target and alarm thresholds that were set through informal expert consultations (with selected scientists only) when no literature data was available are not yet fully informative for a future implementation of the method. This process will be repeated in a follow-up project and a formal expert consultation with a broader panel of experts and stakeholders will be conducted. To further enhance the performance of the developed risk-index model, inclusion of additional data sources should be evaluated. In a next step, the model should be validated in the field, and the applied threshold for a farm being considered “at risk” (deficient in ≥ 50% of control points) can be adjusted accordingly to further increase the sensitivity of the model. The outcomes demonstrated that models using machine-learning algorithms and integrated data sets achieve high sensitivities and can thus serve as a useful tool to support the planning of risk-based animal welfare inspections.

## Conclusions

In a uniform systematic process across the main livestock species in Switzerland, a common method was developed that combines data-based and on-farm indicators to assess animal health and welfare. The results show that proxy data can achieve high correlations with animal health and welfare assessed on-farm, and that using data-based indicators allows to estimate the health status of a farm to a certain extend. However, for a valid assessment, a combination of data-based indicators and on-farm assessments is needed. In order to make on-farm data collection more efficient, PLF technologies could be increasingly used in future. Nevertheless, further research is needed on the validity of indicators monitored by PLF technologies for assessing health and welfare. In addition, existing data sources need to be made more broadly available, as well as linked and interconnected to a higher degree in order to exploit their full potential.

## Data availability statement

The raw data supporting the conclusions of this article will be made available in anonymized form by the authors. Access to public data is regulated by public law. Requests to access these datasets should be directed to the Federal Food Safety and Veterinary Office.

## Ethics statement

The animal study was reviewed and approved by Cantonal Veterinary Offices and the Ethical Committee for animal experiments. Written informed consent was obtained from the owners for the participation of their animals in this study.

## The Smart Animal Health Consortium

The members of the Smart Animal Health consortium are: Joan-Bryce Burla, Lisa Crump, Thomas Echtermann, Sabine G. Gebhardt-Henrich, Nina Keil, Dolf Kümmerlen, Thibault Kuntzer, Isabel Lechner, Rita Lüchinger, Barbara Lutz, Mireille Meylan, Adrian Minnig, Raymond Miserez, Jürg Moll, Stefan Rieder, Gertraud Schüpbach-Regula, Xaver Sidler, Josie Siegel, Joanna Stachowicz, Adrian Steiner, Dimitri Stucki, Beat Thomann, Christina Umstätter, Michael Weber, Beat Wechsler, Hanno Würbel, Patrik Zanolari, Jakob Zinsstag, Romane Zufferey, and Sibylle Zwygart.

## Author contributions

GS-R, HW, and CU contributed to the conception and design of the study. BT was responsible for project management, data acquisition, and wrote the first draft of the manuscript. TK and BT performed data analyses. GS-R, HW, and BT led the project, while CU, BW, and MM supervised individual parts of the study. All authors contributed to the manuscript revision, read, and approved the submitted version. All members of the SAH consortium contributed to the research outcomes.
